# Cooperative protection against stochastic losses: Experimental evidence on behavioral dynamics

**DOI:** 10.1007/s00191-026-00957-6

**Published:** 2026-04-11

**Authors:** Sonja Köke, Andreas Lange, Andreas Nicklisch

**Affiliations:** 1 Christian-Albrechts-Universität zu Kiel, Kiel, Germany; 2 Department of Economics, University of Hamburg, Hamburg, Germany; 3 Center for Economic Policy Research, University of Applied Sciences of the Grisons, Chur, Switzerland

**Keywords:** Ex post rationality, Experiment, Focusing event, Repeated prisoner’s dilemma, Stochastic damages, C92, H41, Q54

## Abstract

We investigate the dynamics of voluntary cooperation in stochastic social dilemmas. In variants of a repeated four-person prisoner’s dilemma game, we show that cooperation rates are larger and more stable when cooperation affects the probability rather than the size of a damage event. We provide insights into behavioral adaptation: after experiencing a damage event, defecting players are more likely to switch to cooperation, while cooperating players are less likely to continue cooperating. In contrast, the absence of damage reinforces the initially chosen strategies. This behavior is consistent with simple learning dynamics based on ex post evaluations of the chosen strategy.

## Introduction

The world currently faces an enormous number of challenges. Climate change, global pandemics such as COVID-19, large-scale forest fires, and shortages of drinking water, to name but a few, threaten our well-being and way of life. Common element to those challenges is their social dilemma structure. Reducing the carbon footprint of an economy, for instance, faces large free-riding incentives. Land development can exacerbate fire risks and impose hazards on many. The occurrence of such adverse events often has a stochastic nature. While cooperation may reduce the likelihood of damage events and/or their consequences, it cannot rule out their occurrence.

Establishing cooperation under such circumstances is problematic: subjects may provide preparation efforts without seeing direct benefits as no damage event materializes. Conversely, the benefits of (existing or missing) cooperation are typically discussed only when the damage occurs. In this case one may be able to identify that better precautions could have led to less severe damages or a full avoidance of the damage event. As a consequence, the experience of damage events can trigger behavioral reactions: for instance, extreme climate events may increase individual protective actions, even though those might be short-lived (e.g., Meyer, 2012; Konisky et al., 2016; Sisco et al., 2017). Likewise, Sisco et al. (2017) find “the actual experiences of the events being more impactful than the purely descriptive information ...made available by the weather forecasts directly before each event hits” (p. 237). This is why Birkland (2006) speaks of adverse events as “focusing events” which may trigger individual behavioral changes (e.g., Spence et al., 2011) as well as policy changes at the societal level (e.g., Kingdon, 1995).^1^

Such findings of behavioral changes *after* experiencing a damage event are puzzling from a traditional game-theoretic view in the absence of strong income effects as long as the damage event does not generate any new information on future damage realizations. Then, individual precautionary efforts are determined solely by beliefs about their impact on future occurrences of stochastic events. In this paper, we challenge this perspective with a laboratory experiment. We test whether and how the dynamics of cooperative efforts depend on the occurrence of damage events in stochastic social dilemmas. For this, we consider a series of repeated four-person prisoner dilemma games with stochastic payoffs and an unknown end period. Subjects may repeatedly choose to invest in protective actions that benefit the entire group. In the short run (one-shot), subjects have incentives to free-ride on others’ investments, whereas repeated interaction allows for sustained cooperation in subgame-perfect equilibria.

In our main treatment conditions, we distinguish between two qualitatively different channels of cooperative protection: actions may impact (i) the *size* of damages or (ii) the *probability* that damages occur. Underlying both channels is a change in the distribution of damage events. Many real word protective actions may influence both channels (e.g., cooperative protection against large-scale forest fires), while in other cases one of the two channels is predominantly affected (e.g., infection protection to decrease the likelihood of a mass breakout in the early days of the COVID-19 pandemic).^2^ Following this line of arguments, we compare settings in which cooperation either reduces the size of a damage that occurs with a given probability (which equals 1 in *DamRed1* and 0.5 in *DamRed*) or reduces the probability of damages of fixed size (*ProbRed*).^3^

Our experimental results show significant differences between cooperation rates in *DamRed1* and *DamRed* on the one hand and *ProbRed* on the other. Unlike predicted by standard theory, subjects are more likely to cooperate to reduce the probability of the damage, rather than to marginally reduce the size of a certain or stochastic damage. These differences between treatments get more pronounced over time. When cooperation reduces the probability of damage, cooperation rates remain rather stable over the series of interactions. In sharp contrast, cooperation rates decline over time when cooperation reduces the size of a certain or stochastic damage that occurs with fixed probability. Yet, experiencing stochastic damages triggers similar underlying behavioral dynamics across all treatments: following a damage event, (i) non-cooperating players are more likely to switch to cooperation, yet (ii) cooperating players are less likely to continue cooperating. In other words, the absence of damage reinforces the chosen strategies, while the occurrence of damage triggers behavioral readjustment.

We provide a model of hind-side adaptation of behavioral strategies to rationalize these experimental findings. The underlying mechanism is the different interpretations of damage events in the two main stochastic treatments: in *DamRed*, the benefits of cooperation materialize through a reduction in harm for all if the damage occurs. In contrast, if the adverse event occurs in *ProbRed*, the cooperative efforts were not sufficient to avoid the damage and thus appear to have been wasted.

Our paper adds to three streams of the literature. First, following the seminal work by Milinski et al. (2008), a literature on cooperative behavior in stochastic social dilemmas analyzes individual efforts to meet a collective target (e.g., Tavoni et al., 2011; Barrett, 2013), including the investigation of the role of uncertain thresholds levels (e.g., Keser and Montmarquette, 2008; Barrett and Dannenberg, 2012; Dannenberg et al., 2014; Carlsson et al., 2024).^4^ Relatedly, the effect of stochasticity has been investigated in social dilemmas on resource appropriation (e.g., Blanco et al., 2015, 2017) and on trade-offs between adaptation and mitigation investments in repeatedly played social dilemmas (e.g., Hasson et al., 2010, 2012; Blanco et al., 2014, 2016). Our paper adds to this literature by investigating the mechanisms behind the behavioral dynamics induced by the realization of stochastic events, i.e. by explicitly comparing the two channels of reducing damage probabilities vs. damage size.

Second, our paper complements the literature that deals with “self-insurance” and “self-protection” (e.g., Ehrlich and Becker, 1972), which considers protective and preventive actions as either complements or substitutes for market insurance (e.g., Dionne and Eeckhoudt, 1985; Jullien et al., 1999; Briys and Schlesinger, 1990). Polinsky and Shavell (2000) consider optimal liability schemes and show that the optimal fine level may be reduced for risk-averse individuals who are better controlled by a larger detection probability. Lohse et al. (2012) investigate a social dilemma game where investments function as an insurance reducing the size of a loss or as a protection against a loss.^5^ Experimental studies by Bruner (2009) and Friesen (2012) consider individual investments in lotteries varying the size or the likelihood of a reward vs. fine. Our research extends this literature by considering multi-player problems with stochastic damages and by focusing on the dynamics of behavior.

Third, we build on the literature on the evolution of cooperation norms in indefinitely repeated social dilemma games, starting with Roth and Murnighan (1978) and Kandori (1992).^6^ Dal Bò and Fréchette (2011) find that the existence of a cooperative equilibrium may be a necessary (but not sufficient) condition for persistent cooperation. Experimental evidence appears mixed: while Duffy and Ochs (2009) do find cooperation to neither sustain under random matching nor to emerge with experience for fixed pairings, Camera and Casari (2009) find sustained cooperation even in anonymous settings, i.e., with re-matching where subjects do not observe any of the actions outside their own pair. We continue this important stream of the literature by demonstrating mechanisms behind the sensitivity of sustained cooperation in indefinitely repeated stochastic social dilemma settings.

Our findings show important differences between the treatment conditions: while the occurrence of a damage event serves as a focal event and triggers behavioral changes across all stochastic treatments, cooperation rates are significantly higher when cooperation reduces the probability rather than the size of the damage. The behavioral dynamics are consistent with combinations of behavioral motives of anticipated regret (e.g., Loomes and Sugden, 1982; Zeelenberg, 1999; Filiz-Ozbay and Ozbay, 2007) and evolutionary learning dynamics which link back to notions of ex post regret (e.g., Selten and Chmura, 2008; Chmura et al., 2012).

The remainder of the paper is structured as follows: Section 2 describes the experimental setting: after describing the game in Section 2.1, we derive predictions in Section 2.2, before detailing the experimental design in Section 2.3. Experimental results are presented in Section 3. Section 4 briefly discusses our findings, before we conclude in Section 5.

## Experimental design and predictions

### Experimental treatments

We study a repeatedly played simultaneous move four-person prisoners’ dilemma. At the beginning of each period, each player is endowed with *E* tokens. At the end of each period, a damage of *D* tokens occurs with probability *p* and reduces the endowment of each player. Damages are fully correlated across the four players; that is, either all players or no player within a group incur the damage in a given period, and damages are independent over time. With their decisions, players may reduce either the size or the probability of the damage, depending on the treatment.

For this purpose, each player *i* is asked in each period *t* before the damage realizes to choose whether to cooperate ($q_i^t=1$) or defect ($q_i^t=0$).^7^ Cooperation costs the individual player *c* tokens. The sum of cooperators in a group and period is denoted by $Q^t=\sum _{j=1}^n q_j^t=q_i^t+Q^t_{-i}$. The potential damage, $D^{Treat}(Q^t)$, and the probability of its occurrence, $p^{Treat}(Q^t)$ depend on the total cooperation level and differ between treatments (*Treat*). With this, the general payoff structure of individual *i* in period *t* is given by

1 $$\begin{aligned} \pi _i^t (q_i^t,Q^t_{-i},s^t)=E-c q_i^t-s^t D^{Treat}(Q^t) \end{aligned}$$

where $s^t\in \{0,1\}$ reflects the state of nature where the damage has ($s^t=1$) or has not ($s^t=0$) occurred.

In the experiment, we differentiate between four treatments which are calibrated to guarantee equivalence in expected damages, that is, $p^{Treat}(Q^t)D^{Treat}(Q^t)$ is equivalent for all treatments. The specific mapping of $Q^t$ in probability and size of damages is summarized in Table 1 for all treatments.

In the first of two main treatments, denoted as *DamRed*, each player’s cooperation leads to a reduction of the initial damage $D_0$ by the amount *d*, while the initial probability is kept constant at $p_0$. That is, we have $D^{\textit{DamRed}}(Q^t)=D_0 -dQ^t$ and $p^{\textit{DamRed}}(Q^t)\equiv p_0$. In the second main treatment, denoted as *ProbRed*, cooperation leads to a reduction of the initial probability of the damage $p_0$ by the amount *x* for each cooperation decision ($p^{\textit{ProbRed}}(Q^t)=p_0 -xQ^t$) while its level is fixed at $D^{\textit{ProbRed}}(Q^t)\equiv D_0$. In order to be able to investigate the effects of stochastic damage occurrence in both *DamRed* and *ProbRed*, we set $p_0<1$. Furthermore, we set $d p_0=x D_0$ in order to guarantee the equivalence of the expected damages $p_0(D_0-dQ^t)=(p_0-xQ^t)D_0$ across both treatments.

The desired social dilemma structure in expected payoff terms requires $np_0d>c>p_0d$ and $nxD_0>c>xD_0$. In other words, the social expected benefits of one additional player cooperating ($np_0d=nxD_0>c$) exceed the cost of cooperation (*c*), while it does not pay off individually ($c>p_0d=xD_0$). Additionally, we assume that even full cooperation ($Q^t=n$) does not reduce expected damages to zero ($p_0 -nx>0$, $D_0 -nd>0$). We consider this a reasonable description of most stochastic damage events.^8^

Two additional treatments consider the sensitivity of behavior: a third treatment, denoted as *DamRed1*, gets rid of the stochasticity while keeping the damage-reducing nature of cooperation. Here, damages occur with certainty, $p^{\textit{DamRed1}}(Q^t)=1$. In order to guarantee the equivalence of (expected) payoffs with other treatments, we set $D^{\textit{DamRed1}}(Q^t)=p_0 D_0-p_0 dQ^t$.

A fourth treatment provides an important sensitivity check to the information environment as probability and damage reduction treatments differ regarding the player’s ability to assess the counterfactual: after receiving information at the end of each period about the total level of cooperation $Q^t$ and on the occurrence of the damage event ($s^t$), players in *DamRed1* and *DamRed* can calculate the payoff that they would have received if they had changed their own decision $q_i^t$. In contrast, players in *ProbRed* cannot, as they lack the information whether or not their own decision was pivotal for triggering/preventing the damage event. For example, after observing a damage event, a defecting player cannot know if the damage also would have occurred if the player had cooperated individually.^9^ Conversely, when no damage occurred, a cooperating player in *ProbRed* does not know if the player was pivotal in preventing the damage event. In order to control for the impact of players’ being informed about their marginal impact on the payoff, we introduce a fourth treatment condition *ProbRed*$^+$ which is identical with *ProbRed* in the mapping of cooperation into probability and damage, but gives players additional feedback after each period: players are informed whether the damage would have occurred if zero, one, two, three, or all four players had cooperated. Therefore, *ProbRed*$^+$ increases the subjects’ awareness about their decision’s marginal impact on the payoff.

**Table 1:** Summary of damage size $D^{Treat}(Q^t)$ and damage probability $p^{Treat}(Q^t)$ for the respective treatments. Equivalence of expected damages is guaranteed by $d p_0=x D_0$. Parameters in the experiment: $p_0=0.5$, x=0.1, $D_0=20$, d=4.

	Damage size	Damage probability
Treatment	$D^{Treat}(Q^t)$	$p^{Treat}(Q^t)$
*DamRed1*	$p_0 (D_0 -dQ^t)$	1
*DamRed*	$D_0 -dQ^t$	$p_0$
*ProbRed*	$D_0$	$p_0-xQ^t$
*ProbRed*$^+$	$D_0$	$p_0-xQ^t$

In all treatment conditions, we apply a random stopping rule for supergames (e.g., Dal Bò and Fréchette, 2011). Each supergame consists of several periods of the game described above and has a publicly known termination probability δ after each period.^10^ At the beginning of each supergame, players are randomly re-matched into new groups. The number of supergames is unknown to the players, and they cannot predict when any given supergame terminates. In order to increase the statistical power, the random draws determining the lengths of the supergames are taken once and applied to all sessions and treatments.

We set the experimental parameters as follows: termination probability δ=0.2, initial damage probability $p_0=0.5$, probability reduction x=0.1, initial damage size $D_0=20$, damage reduction d=4, initial endowment E=25 and cost c=5, thus also ensuring nonnegative period payoffs ($E-cq_i^t-s^tD^{Treat}(Q^t)\ge E-c-D_0=0$). Importantly, we show below that these parameters ensure that cooperative equilibria exist for risk-neutral players, which Dal Bò and Fréchette (2011) suggest is a necessary (but not sufficient) condition for persistent cooperation.

### Predictions

It is obvious that the game has a subgame perfect equilibrium in which *all* players *always* defect: as in the one-shot prisoner’s dilemma game, no player individually has an incentive to cooperate. Yet, the repeated nature of the game with uncertain termination also allows for cooperative equilibria. The proof rests on the assumption that Q≤n players follow a modified grim trigger strategy: they cooperate as long as at least Q-1 other players cooperate, otherwise they defect in all subsequent periods. The remaining n-Q players always defect. This modified grim-trigger strategy calls for infinite punishment following a unilateral defection. It thereby introduces the highest possible costs for deviating. As a consequence, the analysis of grim-trigger shows us the least restrictive condition for cooperative equilibria to exist. In Appendix A, we show that such cooperative equilibrium requires a minimum number of (conditionally) cooperating players:

2 $$\begin{aligned} Q\ge Q^{min}= \frac{c}{p_0d(1-\delta )}-\frac{\delta }{1-\delta }. \end{aligned}$$

For our parameters, $Q^{min}=2.875$, such that at least three players have to cooperate.^11^ Clearly, this condition holds for all treatments since the treatments coincide in terms of mapping actions into expected payoffs. The assumption of risk-neutral decision-makers can thus not provide guidance on potential treatment differences.

Treatment differences may occur if subjects are risk-averse or risk-loving. In Appendix A, we consider players with CRRA risk-attitudes and derive and discuss the conditions under which the modified grim trigger strategy can sustain subgame-perfect equilibria with positive cooperation levels for the respective treatments.

While this can lead to different expectations regarding the chances of sustained cooperation across treatments, none of these derivations from subgame-perfect equilibria can provide predictions about how the experience of damage affects the evolution of strategy choices. Clearly, stochastic damage treatments allow for additional strategies in which players may condition their actions or changes in actions on the occurrence or non-occurrence of a damage event. One could thus construct equilibria such that, ex post, many observed a fitting equilibrium strategy profile could rationalize behavioral changes. However, given the multiplicity of subgame-perfect equilibria and the lack of an intuitive way to select among them, such an ad hoc approach provides no guidance for hypotheses on behavioral changes over time within a given treatment or across treatments. Our experiments thus challenge the view that behavior is explained solely by considering subgame-perfectness. We will provide a complementary explanation in Section 4.

### Experimental procedure

In total, we ran 12 experimental sessions between January and March 2014 at the Experimental Laboratory of the School of Business, Economics and Social Sciences at the University of Hamburg.^12^ Three sessions were conducted for each of the treatment conditions that we described in Section 2.1. A total of 280 students from the University of Hamburg participated in the experiment, with 72 in *ProbRed*, *DamRed*, and *DamRed1*, and 64 in *ProbRed*$^+$. A maximum of 24 and a minimum of 16 subjects participated in an individual session. The median age was 24 years, and 53% were female participants.

We applied the same sequence of periods and supergames across all sessions and treatments, which we randomly determined by computer draws prior to the first experimental session. Overall, all participants played seven supergames; the supergames consisted of 5, 3, 7, 4, 7, 3 and 5 periods, respectively. We organized the rematching at the end of each supergame such that two new groups were randomly formed from a matching unit of eight participants, which remained constant for the entire duration of the session. This gave us nine independent observations in *ProbRed*, *DamRed*, and *DamRed1*, as well as eight independent observations in *ProbRed*$^+$.

After the main experiment, we assessed participants’ risk preferences following Eckel and Grossman (2008) and Dave et al. (2010) with an average payoff of 38 cents (a minimum of 2 cents, and a maximum 70 cents), before adding some brief questions regarding the socio-demographic characteristics of our participants (e.g., gender, age, and years of study).

During the experiment, participants played for taler. At the end of the experiment, the sum of the payoffs across all rounds was converted into euros at an exchange rate of 1 taler for 1 euro cent and paid out privately. Subjects earned an average of 10.50 euros in the repeated prisoners’ dilemma part, with a maximum of 12.70 euros and a minimum of 8.25 euros. Each session lasted for about 60 min. The experiment was programmed and conducted with the software z-Tree (Fischbacher 2007) and recruitment took place with hroot (Bock et al. 2014). The instructions (translated from German to English) can be found in Appendix D.

## Results

We structure our discussion of the results by first considering average treatment differences, before explicitly exploring the role of risk aversion and individual adaptation dynamics after damage events.

### Average treatment differences

**Fig. 1:**
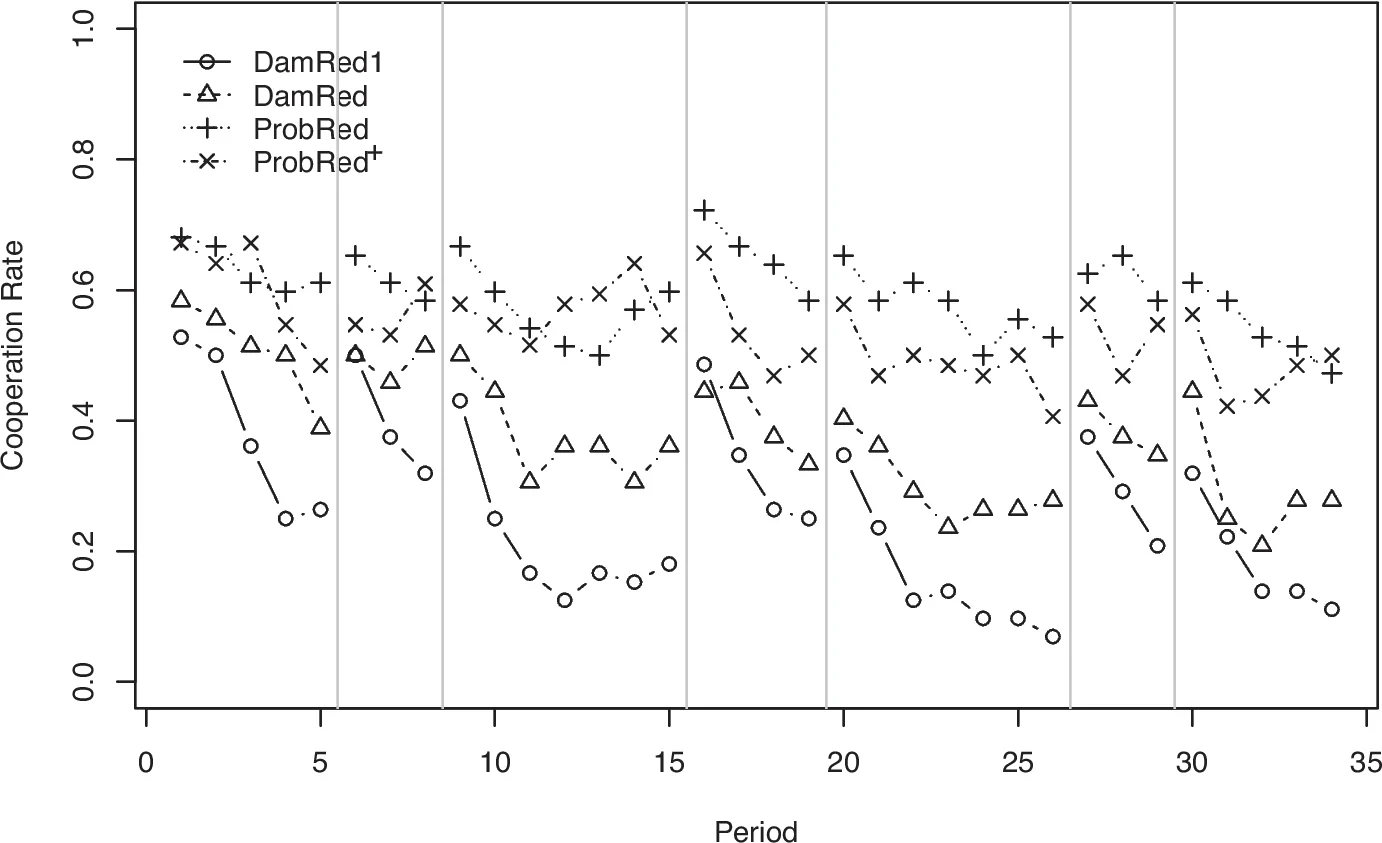
Mean cooperation frequency per period by treatment; *vertical gray lines* indicate the start of new supergames

Figure 1 shows the mean cooperation rates per period and treatment. Table 2 summarizes the average cooperation rates across all periods as well as for the first and last periods of the supergames, and reports *p* values of the respective pairwise treatment comparisons.^13^

**Fig. 2:**
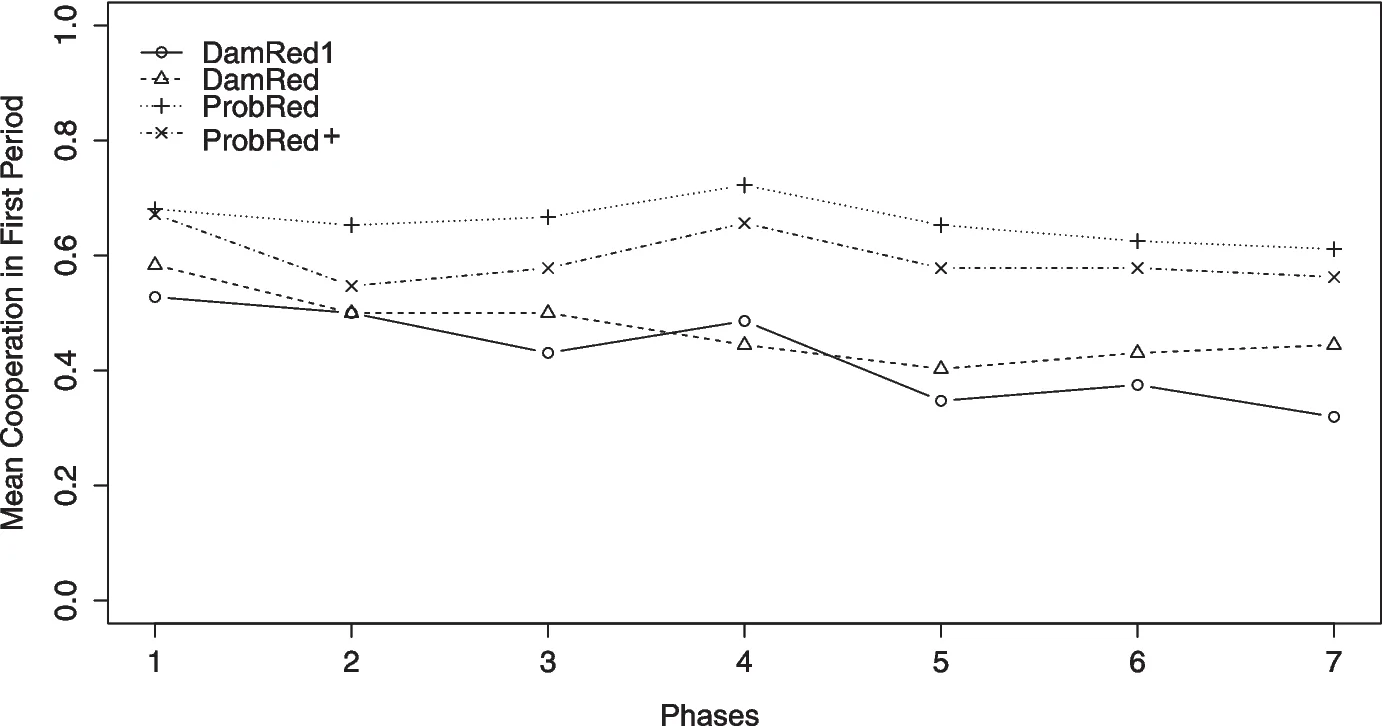
Mean cooperation in the first period of all supergames across treatment conditions

Cooperation rates across all periods are 59% in *ProbRed*, 54% in *ProbRed*$^+$, 38% in *DamRed*, and 26% in *DamRed1*. With this, cooperation rates in *ProbRed* and *ProbRed*$^+$ are substantially higher than in *DamRed* and *DamRed1* (p<0.01). The treatment differences occur qualitatively already in the very first period of the experiment (68% cooperate in *ProbRed*, 67% in *ProbRed*$^+$, 58% in *DamRed*, and 53% in *DamRed1*), but are further strengthened over time. This is also illustrated in Fig. 2, which shows cooperation rates in the first period of the respective supergames. We find a negative trend of cooperation rates in the first periods of supergames in *DamRed* and *DamRed1* (both p=0.05, based on Cuzick’s non-parametric test for trends), while the negative trend is not significant for the probability reduction treatments (p=0.19 and p=0.13, respectively).

**Table 2:** Average cooperation rates (standard deviations) by treatments over the entire experiment (*left panel*), over the first periods of all supergames (*middle panel*), and over the last periods of all supergames (*right panel*). Each matching unit is taken as an independent observation. Tests refer to two-sided Wilcoxon Mann–Whitney rank-sum tests, $$^{***}$$ indicates significance at a p<0.01 level, $$^{**}$$ at a p<0.05 level and $$^{*}$$ at a p<0.1 level

	Average cooperation rate ($q_i^{t}=1$)		
	all periods	first periods	last periods
(1) *DamRed1*	0.26 (0.13)	0.43 (0.13)	0.20 (0.15)
(2) *DamRed*	0.38 (0.14)	0.47 (0.15)	0.36 (0.17)
(3) *ProbRed*	0.59 (0.13)	0.66 (0.11)	0.57 (0.14)
(4) *ProbRed*$^+$	0.54 (0.15)	0.60 (0.18)	0.51 (0.16)
(2) vs. (1)	p=0.130	p=0.619	$>^{*}$, p=0.078
(3) vs. (1)	$$>^{***}$$, p=0.007	$$>^{***}$$, p=0.002	$$>^{***}$$, p<0.001
(4) vs. (1)	$$>^{***}$$, p=0.002	$$>^{**}$$, p=0.033	$$>^{***}$$, p<0.001
(3) vs. (2)	$$>^{***}$$, p=0.007	$$>^{**}$$, p=0.013	$$>^{**}$$, p=0.011
(4) vs. (2)	$$>^{***}$$, p<0.001	$$>^{*}$$, p=0.076	$$>^{*}$$, p=0.079
(4) vs. (3)	p=0.321	p=0.286	p=0.436

Table 3 reports further evidence for the cooperation trends based on a random-effects regression of the individual cooperation decision on the supergame (*supergame*, ranging from 1 to 7) and the period within a supergame (*period in supergame*, ranging from 1 to 7) as well as on dummies for the treatments and the corresponding interaction terms. We use the Bonferroni correction of *p* values for all interacted variable and interaction terms to avoid the multiple testing problem (i.e., the accumulation of the alpha error). We find negative time trends within supergames in all treatments and across supergames in *DamRed* and *DamRed1*, while the trend in *ProbRed* is significantly less negative.^14^ The downward trend within supergames is largest in *DamRed1*, significantly smaller in both *DamRed* and *ProbRed* and weakest in *ProbRed*$^+$.

**Table 3:** Random-effects linear regression of time trends for individual cooperation decision $$q_i^t$$; coefficients are reported along standard errors in parenthesis (errors are clustered at the matching group level); $$^{***}$$ indicates significance at a p<0.01 level, $$^{**}$$ at a p<0.05 level and $$^{*}$$ at a p<0.1 level, Bonferroni correction of *p* values for all interacted variable and interaction terms. *obs* reports the number of observation while *n* report the number of subjects; model’s fitness is assessed by a Wald-Chi$$^2$$-test

	dependent variable: $q_i^{t}$
*DamRed*	0.06 (0.06)
*ProbRed*	$$0.147^{**}$$ (0.06)
*ProbRed*$^+$	0.099 (0.06)
*supergame*	$$-0.031^{***}$$ (0.004)
*supergame* × *DamRed*	$$-0.004^{**}$$ (0.01)
*supergame* × *ProbRed*	$$0.02^{***}$$ (0.005)
*supergame* × *ProbRed*$^+$	0.01 (0.005)
*period in supergame*	$$-0.055^{***}$$ (0.004)
*period in supergame* × *DamRed*	$$0.025^{***}$$ (0.006)
*period in supergame* × *ProbRed*	$$0.033^{***}$$ (0.006)
*period in supergame* × *ProbRed*$$^+$$	$$0.043^{***}$$ (0.006)
*constant*	$$0.558^{***}$$ (0.043)
*obs*	9520
*n*	280
Wald-Chi$$^2$$-test	$$523^{***}$$

Summarizing our first result, we formulate:

#### Result 1

*Cooperation rates are larger when cooperation affects the probability of a damage event* (*ProbRed* *and* *ProbRed*$^+$) *rather than affecting the size of a stochastic damage* (*DamRed*) *or when it leads to a certain damage reduction* (*DamRed1*).

Result 1 conflicts with our prediction, suggesting more cooperation in *DamRed1* than under stochastic damages. The substantially larger cooperation rates in *ProbRed* than in *DamRed* are also not in line with the predictions. Notice, however, that due to bad luck in the randomization of risk attitudes across treatment conditions, we find a significantly higher risk tolerance in *DamRed* than in *DamRed1* and *ProbRed*. Therefore, we proceed with relating risk attitudes and individual decisions to cooperate.

### Individual risk attitudes and cooperation

Table 4 reports the distributions of individual risk tolerance by treatment conditions (measured on a scale from 1 – very risk-averse – to 6 – very risk-seeking). We code subjects as risk-averse (RA) if they choose 1 through 4 and as non-risk-averse if they choose 5 or 6. Risk-aversion levels in *DamRed* are substantially smaller than in *DamRed1* and in *ProbRed*: while in the former 47% are risk-averse, the proportion is 68% in *DamRed1* and 67% *ProbRed*, and 61% in *ProbRed*$^+$.

**Table 4:** Number of subjects with respective risk tolerance measured in the second part of the experiment on a scale from 1 (very risk averse) to 6 (very risk seeking) by treatment condition. Risk-averse type (RA) is coded if choices are 1 through 4, non-risk-averse (NRA) if choices are 5 or 6

	1	2	3	4	5	6	mean (std)	RA	NRA	*n*
*DamRed1*	9	15	15	10	4	19	3.58 (1.77)	49	23	72
*DamRed*	7	6	11	10	21	17	4.15 (1.62)	34	38	72
*ProbRed*	7	13	23	5	9	15	3.57 (1.65)	48	24	72
*ProbRed*$^+$	12	7	12	8	10	15	3.65 (1.82)	39	25	64
*All treatments*	35	41	61	33	44	66	3.74 (1.72)	170	110	280

Table 5 reports results from both regressions analyzing decisions in the first period of the first supergame as well as from individual random effect models (with errors clustered at the matching group level) analyzing decisions across all periods and all supergames. For both, we control for risk aversion and report the regressions separately for risk-averse (RA) and non-risk-averse (NRA) subjects. None of the interaction terms between NRA and treatments is significant (Bonferroni correction of *p* values applied for all interacted variables and interaction terms), suggesting that the individual decisions do not significantly depend on the individual’s risk aversion.^15^ The treatment effects between the damage reduction and probability reduction treatments remain robust.

**Table 5:** *Left panel* ((1)–(3)): linear regression of cooperation behavior in the first period, *right panel* ((4)–(6)): individual random effects regression of cooperation behavior in all periods of the experiment; coefficients are reported along standard errors in parenthesis (errors are clustered at the matching group level); $$^{***}$$ indicates significance at a p<0.01 level, $$^{**}$$ at a p<0.05 level and $$^{*}$$ at a p<0.1 level; Bonferroni correction applied of *p* values applied for all interacted variable and interaction terms. *obs* reports the number of observations while *n* reports the number of subjects

	(1)	(2)	(3)	(4)	(5)	(6)
	dependent variable: $q_i^{t}$			dependent variable: $q_i^{t}$		
	only first period			all periods		
	all	only RA	only NRA	all	only RA	only NRA
*DamRed*	0.04	0.04	0.02	0.08	0.08	$$0.18^{**}$$
	(0.109)	(0.109)	(0.129)	(.076)	(0.08)	(0.068)
*ProbRed*	$$0.20^{*}$$	$$0.20^{**}$$	0.06	$$0.33^{***}$$	$$0.33^{***}$$	$$0.34^{***}$$
	(0.099)	(0.099)	(0.142)	(0.81)	(0.08)	(0.079)
*ProbRed*$$^+$$	0.18	$$0.18^{*}$$	0.07	$$0.25^{***}$$	$$0.25^{***}$$	$$0.33^{***}$$
	(0.105)	(0.105)	(0.141)	(0.083)	(0.084)	(0.075)
*NRA*	0.12			-0.05		
	(0.123)			(0.07)		
*NRA*×*DamRed*	-0.02			0.09		
	(0.169)			(0.09)		
*NRA*×*ProbRed*	-0.14			0.01		
	(0.174)			(0.12)		
*NRA*×*ProbRed*$^+$	-0.11			0.08		
	(0.176)			(0.10)		
*constant*	$$0.49^{***}$$	$$0.49^{***}$$	$$0.61^{***}$$	$$0.28^{***}$$	$$0.28^{***}$$	$$0.23^{***}$$
	(0.49)	(0.07)	(0.101)	(0.05)	(0.054)	(0.036)
*obs*	280	170	110	9520	5780	3740
*n*	280	170	110	280	170	110

### Individual response to focusing events

We proceed with investigating how the experience of damage events may trigger behavioral changes at the individual level. By construction of our treatments, the frequency with which the damage occurs is significantly lower in *ProbRed* and *ProbRed*$^+$, 0.28 and 0.31, respectively, than in *DamRed*, 0.48.^16^. Thus, subjects in the former two treatment conditions experience a focusing event at a substantially lower frequency than subjects in the latter treatment condition.

In a first step, we consider the conditional frequencies of $q_i^{t+1}=1$ given $q_i^{t}$ and the occurrence of the damage $s^t$. Table 6 summarizes the frequencies by treatment conditions as well as the treatment comparisons based on non-parametric Mann–Whitney tests.

**Table 6:** Mean $$q_i^{t+1}$$ (std) given $$q_i^{t}$$ and the occurrence of the damage $$s^t$$. The reported numbers are the means (standard deviation) of cooperation levels averaged over matching units. Tests refer to the corresponding two-sided Wilcoxon Mann–Whitney rank-sum tests, $$^{***}$$ indicates significance at a p<0.01 level, $$^{**}$$ at a p<0.05 level and $$^{*}$$ at a p<0.1 level

	Average cooperation rate ($q_i^{t+1}=1$) in period t+1 within matching unit			
	conditional on $q_i^{t}=0$ and		conditional on $q_i^{t}=1$ and	
	$s^t=1$	$s^t=0$	$s^t=1$	$s^t=0$
(1) *DamRed1*	0.14 (0.07)	−	0.54 (0.15)	−
(2) *DamRed*	0.17 (0.05)	0.19 (0.10)	0.61 (0.16)	0.73 (0.15)
(3) *ProbRed*	0.24 (0.12)	0.13 (0.08)	0.80 (0.08)	0.89 (0.07)
(4) *ProbRed*$^+$	0.28 (0.15)	0.33 (0.18)	0.66 (0.25)	0.72 (0.19)
(2) vs. (1)	p=0.258	−	p=0.258	−
(3) vs. (1)	$$>^{**}$$, p=0.024	−	$$>^{***}$$, p<0.001	−
(4) vs. (1)	$$>^{**}$$, p=0.021	−	p=0.200	−
(3) vs. (2)	p=0.258	p=0.297	$$>^{***}$$, p=0.006	$$>^{***}$$, p=0.003
(4) vs. (2)	$$>^{**}$$, p=0.027	$$>^{**}$$, p=0.027	p=0.370	p=0.762
(4) vs. (3)	p=0.497	$$>^{***}$$, p=0.002	p=0.321	$$<^{***}$$, p=0.006

Comparing *ProbRed* versus *DamRed*, we find a more stable cooperation in the former among those players who already cooperate. The additional information on pivotality on triggering/preventing the damage event in *ProbRed*$^+$, leads more defecting players to switch to cooperation than in *DamRed*. Notice that *ProbRed*$^{+}$ appears to lead to different individual dynamics compared to *ProbRed*. In particular, when the damage did not occur, the frequency of cooperation in t+1 for initial non-cooperators is substantially larger in *ProbRed*$^{+}$ than in *ProbRed*, while the opposite holds for those who already cooperated in period *t*.^17^

For a detailed analysis of individual learning in our game, we estimate a series of Arellano–Bond panel regressions, for each treatment condition separately.^18^ This allows us to analyze endogenous regressors (see Arellano and Bond, 1991): the dependent variable is $q_i^{t+1}$ (i.e., the decision whether to cooperate or defect in the consecutive period). As explanatory variables, we use $Q_{-i}^t$ (i.e., the number of cooperators except *i* in the current period), the occurrence of the damage in *t* (i.e., we compute a dummy variable $s^t$, which is 1 if the damage occurred in *t* and 0 otherwise; omitted in *DamRed1*), $q_i^{t}$ (i.e., the decision whether to cooperate or defect in the current period), and interaction terms $q_i^{t}\times s^t$, as well as $Q_{-i}^t\times s^t$. Furthermore, we control for the beginning of a new supergame (i.e., the dummy variable *newsupergame* is 1 in the first period of a new supergame and 0 otherwise). Again, to prevent the multiple testing problem, we use the Bonferroni correction of *p* values for all interacted variables and interaction terms.

To access the additional information provided in *ProbRed*$^{+}$, we additionally introduce a variable measuring the number of cooperators exceeding the necessary number to avoid the realization of the damage. That is, the variable Δcooperator computes the difference between the actual players cooperating and the cooperators required by nature for the absence of damage. Δcooperator is zero if the number of cooperators just coincides with the number required to avoid the damage, it is negative if too few players cooperate to prevent the damage and is positive if even a smaller number of cooperators were necessary to prevent the damage. Hence, we test whether players coordinate their cooperation onto a sufficient number of cooperators in the previous period. Estimations for coefficients along with standard errors in parentheses are reported in Table 7.

**Table 7:** Estimation results for an Arellano–Bond panel regressions with dependent variable $$q_i^{t+1}$$; coefficients are reported along standard errors in parenthesis; $$^{***}$$ indicates significance at a p<0.01 level, $$^{**}$$ at a p<0.05 level and $$^{*}$$ at a p<0.1 level, Bonferroni correction of *p* values for all interacted variable and interaction terms. Standard errors are clustered at the matching group level. *obs* reports the number of observation while *n* reports the number of subjects; models’ fitness are assessed by Wald-Chi$$^2$$-tests

	dependent variable: $q_i^{t+1}$				
	*DamRed1*	*DamRed*	*ProbRed*	*ProbRed*$^+$	*ProbRed*$^+$
$$q_i^{t}$$	$$.219^{***}$$ (.022)	$$.368^{***}{^{*}}$$ (.03)	$$.29^{***}{0}$$ (.027)	$$.066^{*}{^{*}}$$ (.028)	$$.094^{***}$$ (.029)
$$Q_{-i}^t$$	$$.083^{***}$$ (.012)	$$.043^{**}$$ (.016)	$$.028{^{***}}$$ (.013)	$$.06^{***}$$ (.016)	$$.096^{***}$$ (.018)
$$s^t$$		$$.106^{***}{^{*}}$$ (.03)	$$.113^{***}$$ (.036)	$$.052{^{***}}$$ (.043)	$$-.086{^{***}}$$ (.055)
$$q_i^{t}\times s^t$$		$$-.395^{***}$$ (.038)	$$-.26^{***}{0}$$ (.032)	$$-.183^{***}$$ (.042)	$$-.190^{***}$$ (.042)
$$Q_{-i}^t\times s^t$$		$$.017{^{****}}$$ (.02)	$$.017{^{***}}$$ (.018)	$$.006{^{***}}$$ (.023)	$$-.001{^{***}}$$ (.023)
*newsupergame*	$$-.034^{*}{^{**}}$$ (.020)	$$-.005{^{****}}$$ (.020)	$$.011{^{***}}$$ (.016)	$$-.06^{***}{^{*}}$$ (.022)	$$-.063^{***}$$ (.021)
Δcooperator					$$-.041^{***}$$ (.011)
*const*	$$.130^{***}$$ (.012)	$$.194^{***}$$ (.023)	$$.360^{***}$$ (.029)	$$.406^{***}$$ (.033)	$$.412^{***}$$ (.033)
*obs*	2232	2232	2232	1984	1984
*n*	72	72	72	64	64
Wald-Chi$$^2$$-test	$$191^{***}$$	$$183^{***}$$	$$9841^{***}$$	$$50^{***}$$	$$63^{***}$$

The estimation results in Table 7 confirm our previous findings in Table 6. They indicate that cooperation is highly path dependent in all treatment conditions: if a player cooperates in period *t*, it is very likely that the player cooperates in period t+1 as well (significant positive marginal effect of $q_i^{t}$). For all treatments, we also find evidence that subjects reciprocate on others’ cooperation (significant positive coefficients for $Q_{-i}^t$).

Importantly, experiencing a damage event triggers behavioral changes: non-cooperators are more likely to switch to cooperation following a damage event in both *ProbRed* and *DamRed* (positive coefficients for $s^t$, p<0.05). The impact of damage events on the cooperation decision of cooperators is significantly different (negative coefficients for the interaction $q_i^{t}\times s^t$, p<0.01). In fact, a damage event significantly reduces the likelihood that cooperators will continue cooperating (p < 0.01, based on analogous Arellano-Bond panel regressions with cooperators as the basis). When information is given on the cooperation rate needed for preventing the damage event in *ProbRed*$^+$, the significant negative coefficient of Δcooperator suggests that players condition their cooperativeness on the number of cooperators needed to prevent the damages in the previous period: if there are more (less) players than needed to avoid the damage, the likelihood to cooperate decreases (increases).

#### Result 2

In all treatment conditions with stochastic payoffs, the occurrence of damage stimulates a strategy switch of players from defection to cooperation and from cooperation to defection, while the non-occurrence of the damage reinforces existing strategy choices.

Result 2 showcases the importance of focusing on events. Experiencing the damage leads to behavioral adjustments: players condition their behavior (partly) on the occurrence of the random event. Their current strategies are reinforced after experiencing the absence of damage. In contrast, cooperators in *ProbRed* and *ProbRed*$^+$ appear to regret their action when damage has occurred and are more likely to switch towards defection. Surprisingly, however, we observe the same behavioral change in *DamRed* where again cooperators are more likely to switch towards defection following a damage event.

## Discussion

Our experiment gives behavioral evidence that adverse events can lead to behavioral adjustments and thus function as focusing events. This insight has substantial implications for the theoretical analysis of stochastic dilemmas. The traditional approach of forward-looking elaboration presented above results provides little guidance to the level of cooperation or to the dynamics of decisions in the respective treatments. In contrast, the evidence suggests that subjects evaluate their strategies backward-looking: they adjust their strategies depending on the realization of damages, even though this does not provide new information regarding possible future adverse events.

We provide a formal analysis of a hind-side evaluation and adjustment of behavioral strategies in social dilemmas with stochastic payoffs in Appendix B. The underlying mechanism states that a strategy is more frequently chosen if it performed relatively well in the past.^19^ The model predicts that cooperation is not phased out in the long run when cooperating over probabilities (i.e., in *ProbRed* and *DamRed*), while cooperation disappears over periods when it solely affects the size of damages (in *DamRed* and *DamRed1*). With this, such a model can rationalize the different levels of cooperation that we identified in Result 1 and the general time trend of average cooperation levels.

The general intuition for this result comes from the fact that defection is a dominant strategy in the stage game in *DamRed1* and *DamRed* even when knowing realization of $s^t$. However, in *ProbRed* and *ProbRed*$^+$, a player may be pivotal in avoiding the damage, so that cooperation gets reinforced with a positive probability: pivotal cooperating players are more likely to continue choosing cooperation when no damage occurs, whereas defecting players may regret their wrong choice in hind-side if a damage occurs.^20^ This logic is consistent with the behavioral adjustments formulated in Result 2. We thus conclude that such backward-looking strategy adaptation is a useful approach for formally conceptualizing behavior in the aftermath of focusing events.

## Conclusion

This paper investigates determinants of cooperation and its evolution in repeated social dilemmas with stochastic damages. Handling Internet security, preparations for natural disasters like earthquakes, or taking precautions against global pandemics like COVID-19 are just some examples of this class of problems. Despite its widespread prevalence, little is known about the determinants of how individuals behave in such stochastic social dilemmas or how they adjust their strategies over time. To address these questions, we employ a multiplayer prisoner’s dilemma with stochastic damages and an unknown duration of repeated play.

We compare treatments where cooperation either reduces the size of damages or reduces the probability that damages occur. This distinction turns out to be crucial: cooperation rates are larger and more stable over time when cooperation affects the probability rather than the size of a damage event. Furthermore, we show that experiencing a damage event leads to behavioral adjustments: while defectors are more likely to switch towards cooperation after an adverse event, formerly cooperating players may switch towards defection. These results indicate that individuals evaluate and adjust their behavioral strategies based on a backward-looking logic.

Our findings thus provide laboratory experimental evidence that adverse events can indeed serve as focusing events (e.g., Kingdon, 1995; Birkland, 2006).^21^ Unlike earlier studies on single decision maker investments in lotteries (e.g., Bruner, 2009), our results on cooperation within groups indicate a rather minor importance of individual risk attitudes. Consistent with findings by Dal Bò and Fréchette (2011), our findings suggest that the existence of the cooperative equilibrium is not a sufficient condition for persistent cooperation in the group context. Rather, the exact implementation of stochasticity matters: it is crucial whether cooperation affects the size of an adverse event or its probability. In the latter, joint cooperation reinforces itself through the absence of the damage event, thus leading to significantly higher cooperation rates than when cooperation affects the size of damages only.

Overall, our results offer cautiously optimistic prospects for voluntary cooperation in stochastic social dilemma situations: more sustained cooperation can be expected when cooperative behavior has the potential to avert an adverse event that would otherwise lead to a discrete payoff change. While the findings from our laboratory experiment should be interpreted with caution in relation to our motivating examples, they may nevertheless provide insight into the mechanisms underlying successful cooperation. It appears worthwhile to further investigate the robustness of these results across different real-world settings.

## Figures and Tables

**Fig. 3:**
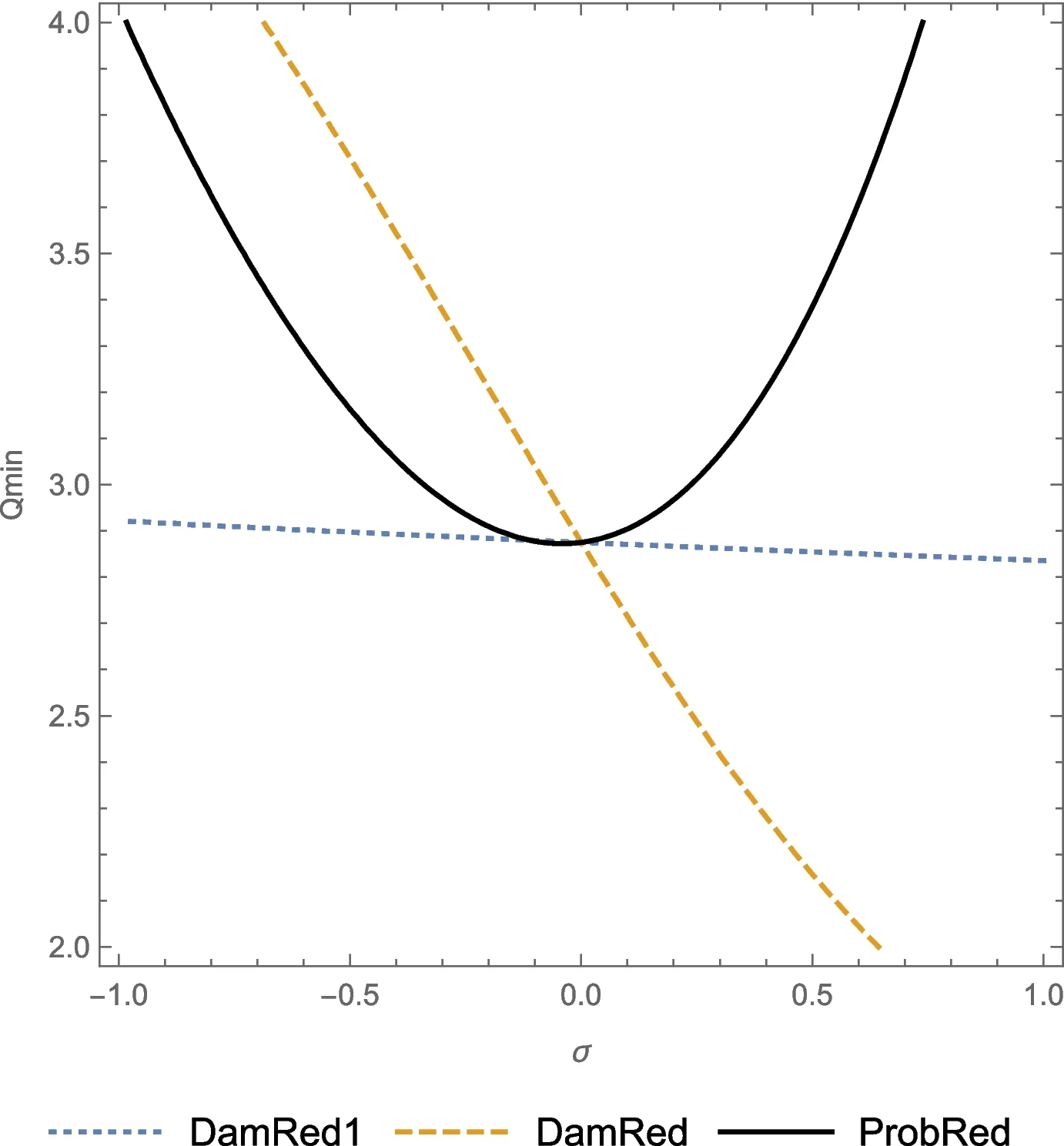
Minimal cooperation level $Q^{min}$ required to stabilize cooperation as a function of risk aversion σ for CRRA preferences ($u(\pi )=\pi ^{1-\sigma }/(1-\sigma )$). Parameters as used in experiment (δ=0.2, $p_0=0.5$, x=0.1, $D_0=20$, d=4, E=25, c=5)

**Fig. 4:**
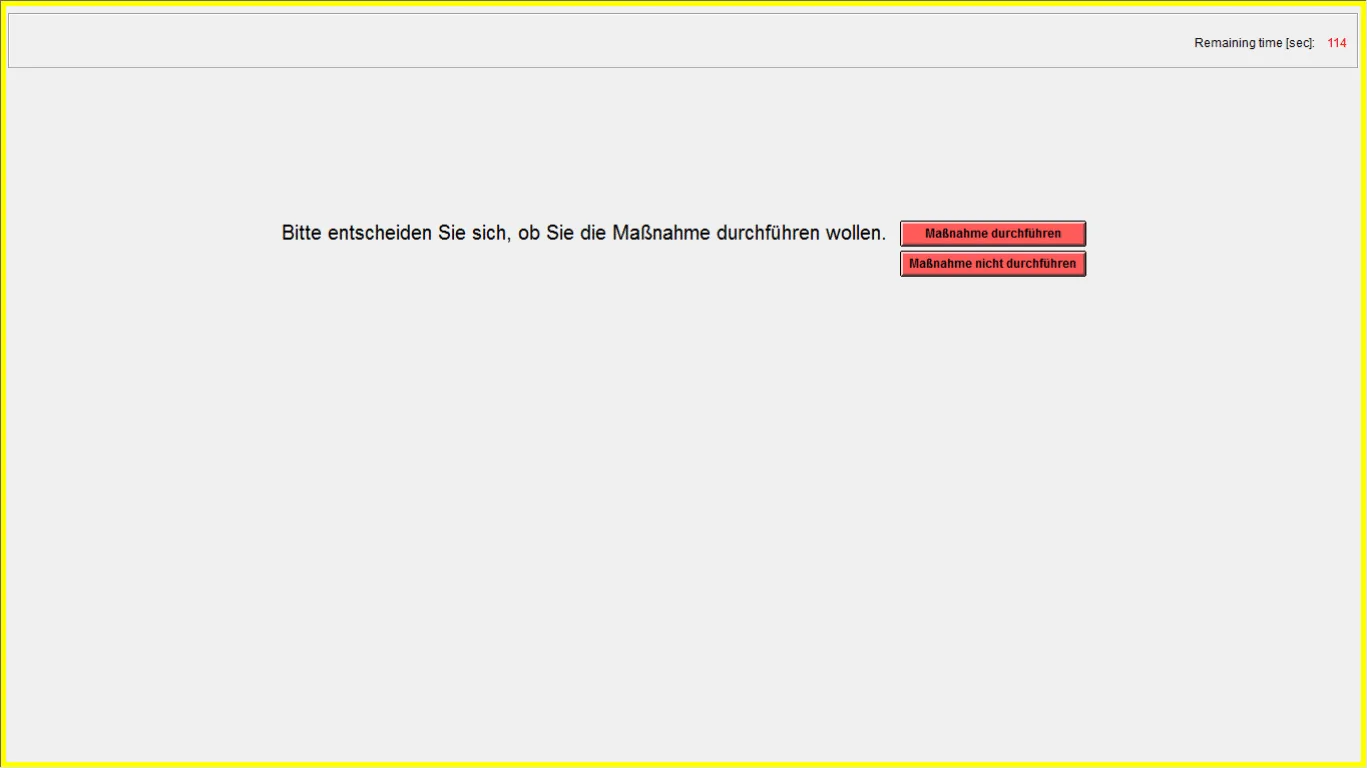
Decision screen for taking the action in a round in Part 1 of the experiment

## Data Availability

The complete data of the experiment are available at: https://osf.io/hr42k/?view_only=47e8b4a1810044b38c0369a24569062f.

## Appendix A: Conditions for subgame-perfect equilibria with sustained cooperation

### Proof of Condition (2) under risk-neutrality

For risk-neutral players, the modified grim-trigger strategy described in Section 2.2 can sustain cooperation as a subgame perfect equilibrium if

A.1 $$\begin{aligned} &  \sum _{t=0}^\infty (1-\delta )^t \left[ E-c-p_0(D_0-d Q) \right] \nonumber \\ &  \ge \left[ E-p_0(D_0-d(Q-1))\right] + \sum _{t=1}^\infty (1-\delta )^t (E-p_0 D_0) \nonumber \\ \Leftrightarrow \quad &  \frac{1}{\delta } \left[ E-c-p_0(D_0-d Q) \right] \nonumber \\ &  \ge \left[ E-p_0(D_0-d(Q-1))\right] + \frac{1-\delta }{\delta } (E-p_0 D_0) \end{aligned}$$

Here, the left-hand side states the expected payoff of any cooperating player *i* if all *Q* players continue to cooperate for an unknown number of periods. The first expression of the right-hand side states the payoff of a deviator *i* in the period in which he deviates, while the second term states the expected continuation payoff if all players play defect, starting in the next period. Given the defection of other players, the deviating player has no incentive to return to cooperation. Therefore, if condition Eq. A.1 is satisfied, *Q* players playing the modified grim trigger strategy and n-Q players always defecting establishes a subgame perfect equilibrium.

Solving condition Eq. A.1 for *Q* immediately leads the condition stated in Eq. 2:

A.2 $$\begin{aligned} Q\ge Q^{min}= \frac{c}{p_0d(1-\delta )}-\frac{\delta }{1-\delta }. \end{aligned}$$

□

### Derivations of conditions for sustained cooperation for CRRA risk attitudes

We assume that players receive a utility from the period payoff $u_i(\pi _i^t)$, where we allow for risk-aversion, i.e., non-linear $u_i(\cdot )$. We rewrite condition Eq. A.1 for the different treatments to see when a player does not have an incentive to deviate from cooperation. We again focus on the assumption that all other cooperating players play a modified grim trigger strategy. For *DamRed1*, a player would keep cooperating if:

A.3 $$\begin{aligned} &  \frac{1}{\delta } \left[ u_i(E-c-p_0(D_0-d Q)) \right] \nonumber \\ &  \ge \left[ u_i(E-p_0(D_0-d(Q-1)))\right] + \frac{1-\delta }{\delta } u_i(E-p_0 D_0). \end{aligned}$$

Here, the left-hand side represents the expected utility under cooperation, given the continuation probability δ, whereas the right-hand side denotes the current-period payoff under unilateral defection together with the discounted expected utility from the continuation game under full defection by all players.

For *DamRed*, continuing cooperation is beneficial if:

A.4 $$\begin{aligned} &  \frac{1}{\delta } \left[ p_0 u_i(E-c-(D_0-d Q)) +(1-p_0) u_i(E-c)\right] \nonumber \\ &  \ge \left[ p_0 u_i(E-(D_0-d(Q-1)))+(1-p_0) u_i(E)\right] \nonumber \\ &  + \frac{1-\delta }{\delta }\left[ p_0 u_i(E-D_0)+(1-p_0) u_i(E)\right] , \end{aligned}$$

while for *ProbRed* we obtain:

A.5 $$\begin{aligned} &  \frac{1}{\delta } \left[ (p_0-xQ) u_i(E-c-D_0) +(1-p_0+xQ) u_i(E-c)\right] \nonumber \\ &  \ge \left[ (p_0-x(Q-1)) u_i(E-D_0))+(1-p_0+x(Q-1)) u_i(E)\right] \nonumber \\ &  + \frac{1-\delta }{\delta }\left[ p_0 u_i(E-D_0)+(1-p_0) u_i(E)\right] . \end{aligned}$$

For each of the treatments, we can define $Q^{min}(\sigma )$ as the value of *Q* that satisfies the respective condition Eqs. A.3, A.4 or A.5 with equality.

As an analytical solution proves impossible, Fig. 3 displays the simulation results for players with constant relative risk aversion (CRRA), i.e., utility $u_i(\pi _i^t)=(\pi _i^t)^{1-\sigma }/(1-\sigma )$. Here, $\sigma \rightarrow 0$ reflects risk-neutrality, while risk-aversion is given for σ>0. Obviously, all the curves collapse for risk-neutral players (σ=0), yielding $Q^{min}=2.875$. It can be seen that for risk-averse decision-makers (σ>0) the threshold $Q^{min}$ is lowest for *DamRed*, while for risk-lovers (σ<0) *DamRed1* mostly leads to the smallest $Q^{min}$.

For *DamRed*, the threshold $Q^{min}$ is decreasing in σ. More risk-averse players are thus willing to be part of a smaller subset of *Q* cooperating players, while very risk-seeking players are not even willing to cooperate if everyone else cooperates. For *DamRed1*, we observe that cooperation is only slightly sensitive to risk attitudes because of the stochastic continuation game. For *ProbRed*, we obtain a U-shaped relationship between $Q^{min}$ and σ in Fig. 3. Here, only players with intermediate levels of risk aversion may cooperate for any given threshold level $Q^{min}$.^22^ Note that for $Q^{min}=2$ this set is empty, while for $Q^{min}=3$ it is fully contained in the set of potentially cooperating players under *DamRed1*. We thus predict cooperation rates to be lower in *ProbRed* than in *DamRed1* if players behave as expected utility maximizers. Cooperative equilibria with two players cooperating may only exist for *DamRed* and only for sufficiently risk-averse subjects.

Provided that the damage creates a loss setting, prospect theory suggests that subjects tend to behave as risk-lovers, such that Fig. 3 suggests that *DamRed* might be least likely to sustain cooperative equilibria. Additionally, probability weighting may apply. In the range of our probabilities in the *ProbRed* (10% to 50%), the typical weighted probability function is rather flat. That is, the marginal impact of cooperation on the weighted probability can be even smaller than the effect on the probability itself. As such, cooperation would pay off even less in *ProbRed*. That is, due to probability weighting in our probability range, we would predict reduced cooperation levels in *ProbRed* than in the other treatments (where the probability is not affected through decisions).^23^

## Appendix B: Backward-looking adaptation of behavior

To formalize the idea that players apply ex post rationality (i.e., adapt their strategies based on the success of their previously chosen), we will apply the impulse balance learning (e.g., Chmura et al., 2012). Formally, there is an initial attraction $A_{i,0}(q)$ of player *i* to play action $q\in \{0,1\}$. Selten and Chmura (2008) assume that the attraction of action *q* evolves according to

B.1 $$\begin{aligned} A_{i,t+1}(q)=A_{i,t}(q)+\max \{0,\pi _{i,t} (q,Q^t_{-i},s^t(q))-\pi _{i,t} (1-q,Q^t_{-i},s^t(1-q))\}, \end{aligned}$$

where $s^t(q)$ ($s^t(1-q)$) denotes the state of the damage event if action *q* (1-q) was chosen. That is, an action is reinforced if it would have been or is the better strategy. The probability of action *q* being played in period t+1 is simply its attraction relative to the sum of the attractions of both actions available to individual *i*:

B.2 $$\begin{aligned} P_{i,t+1}(q)=\frac{A_{i,t}(q)}{A_{i,t}(0)+A_{i,t}(1)}. \end{aligned}$$

Note that the extent of reinforcement in Eq. B.1 equals the payoff difference between both actions. In *DamRed1* and *DamRed*, defection is a dominant strategy: the payoff difference to cooperation is 3 in *DamRed1*, and 1 if a damage occurs or 5 if it does not (i.e., in expectations 3) in *DamRed*. As such, only defection is reinforced in *DamRed1* and *DamRed* (on average by 3 per period). Impulse-balance learning would therefore explain cooperation to be phased out over time at a similar rate in *DamRed1* and *DamRed*:^24^

B.3 $$\begin{aligned} \mathbb {E}[P_{i,t+1}(1)]=\frac{A_{i,0}(1)}{A_{i,0}(0)+3t+A_{i,0}(1)}\rightarrow _{t\rightarrow \infty } 0. \end{aligned}$$

In *ProbRed* and *ProbRed*$^+$, however, player *i* may be pivotal in triggering the damage event. This happens with 10% probability^25^ and would lead to cooperation being the superior action (payoff difference $D_0-c=20-5=15$). With 90% probability, the player cannot affect the damage event, in which case defection ex post would have been the better choice (payoff difference c=5). If players behave according to the correct probability of having been pivotal, cooperation is therefore reinforced in 10% of the periods with a payoff difference of 15, while in the remaining 90% of the cases defection is reinforced by 5. Per period within a supergame, in expectation $A_{i,t}(0)$ grows by 4.5 and $A_{i,t}(1)$ by 1.5, such that the expected probability of cooperation after *t* periods is

B.4 $$\begin{aligned} \mathbb {E}[P_{i,t+1}(1)]=\frac{A_{i,0}(1)+1.5t}{A_{i,0}(0)+A_{i,0}(1)+6t}\rightarrow _{t\rightarrow \infty } 0.25. \end{aligned}$$

Thus, impulse-balance learning suggests that cooperation is not phased out in the long run when cooperating over probabilities. Instead, the likelihood of cooperation converges towards 25%. Note that if a damage event occurs, cooperating players have (ex post) obviously chosen the wrong action, while they may have been right if no damage event occurs. Cooperating players should thus be more likely to switch towards defection after a damage event than when no damage occurred. Conversely, defecting players may have been wrong in their choice if damage occurs, while the absence of the damage event proves that their defection was right. Defectors are therefore predicted to be more likely to cooperate following a damage event than when no damage occurred.^26^

## Appendix C: Analysis of individual risk tolerance measure as dependent variable

Since we measure individual risk attitudes after the main experiment, the risk parameters may also be affected by differences in damage experiences and earnings across treatments. To check for this potential confound, we estimate a regression analysis with individual risk tolerance as the dependent variable and the number of experienced damages and total earnings in the first part of the experiment as independent variables for each treatment condition separately. To increase the power of our analysis, standard errors are bootstrapped based on 10,000 repetitions. The estimation results reported in Table 8 do not indicate any significant influence of the two independent variables on individual risk tolerances in any treatment condition, suggesting that an individual’s risk aversion is not systematically influenced by experience throughout the experiment.

**Table 8:** Linear regression of individual risk tolerance measure (scaled from 1, risk averse, to 6, risk-seeking), estimated for each treatment condition separately; coefficients are reported along standard errors in parenthesis; standard errors are bootstrapped based on 10,000 repetitions; $$^{***}$$ indicates significance at a p<0.01 level, $$^{**}$$ at a p<0.05 level and $$^{*}$$ at a p<0.1 level. *obs* reports the number of observations

	*DamRed1*	*DamRed*	*ProbRed*	*ProbRed*$^+$
	*dependent variable*: individual risk tolerance			
Number of damages		-0.0350	-0.0025	0.0145
		(0.1019)	(0.0643)	(0.0870)
Total sum of incomes	-0.0048	-0.0009	0.0021	-0.0024
	(0.0068)	(0.0048)	(0.0036)	(0.0057)
*constant*	$$ 6.1621^{*}$$	5.2504	2.4364	4.8192
	(3.6910)	(4.0380)	(2.4391)	(3.7389)
*obs*	72	72	72	64
Wald-Chi$^2$-test	0.49	0.12	0.64	0.46

## Appendix D: Experimental instructions for the *DamRed* treatment (English translation)

In the following, we report an English translation of the experimental instructions for the *DamRed* treatment.

General instructions for the participants

You are now taking part in an economic science experiment. If you carefully read the following instructions, you can, depending on your decisions, earn a not inconsiderable amount of money. Therefore, it is very important that you carefully read the following instructions.

The instructions we gave you are intended solely for your private use. During the experiment, communication is completely prohibited. If you have any questions, please raise your hand out of the cabin. Someone will then come to you to answer your question. Violating this rule results in exclusion from the experiment and all payments.

During the experiment, we do not have euros but taler. Your total income will first be computed in taler. The total amount of taler that you earned during the experiment will be converted into euros in the end, such that

100 taler = 1 euro.

At the end of the experiment, you will be paid in cash the total amount of taler that you earned (converted into euros) plus 5 euros for participation. We will conduct the payment such that no other participant will see your payment.

### The experiment is divided into two parts

Here are the instructions for the 1st part. You will get the instructions for the 2nd part on your computer screen after the 1st part is finished. The two parts are not related with respect to their content.

Explanations for the 1st part of the experiment

The 1st part of the experiment is divided into *phases*. You do not know, however, how many phases there are in total. Each phase is divided into *rounds*. The number of rounds in a phase is random. After each round, the phase ends with a probability of 20%.

More concretely, this means that: after the first round, there is a second round with a probability of 80% (which is, on average, in four cases out of five). So, with a probability of 20% (which is on average in one case out of five), the phase ends after the first round. After the second round (if there is one), there is a third one with a probability of 80%. So, with a probability of 20%, the phase ends after the second round and so on.

At the beginning of each phase, participants are randomly assigned to groups of four. Thus, your group has three other members in addition to you. During one phase, the group’s constellation remains unchanged. It only gets randomly rematched at the beginning of a new phase.

Information on the structure of a round

All rounds in all phases are always structured in the exact same way. In the following, we describe the structure of one round.

At the beginning of each round, every participant gets an income of 25 taler.

At the end of each round, damage might occur, reducing income by 20 taler.

The damage occurs with a probability of 50% (which is on average in one of two rounds). For this, in each round, the computer randomly determines whether the damage occurs. The occurrence of the damage is only valid in the respective round and does not influence the probability of the next rounds. The occurrence of the damage is determined jointly for the whole group, such that either all or no group members suffer the damage.

All group members are able to reduce the potential damage through their decisions. For this, at the beginning of each round, i.e., before the damage occurs, each group member has to decide whether it does or does not carry out a damage-reducing action (see Fig. 4).

Each damage-reducing action costs the group member taking the action 5 taler (independent of whether the damage occurs or not). Each damage-reducing action reduces the personal damage of each group member (not only of the group member taking the action) by 4 taler. For you, personally, this means that each damage-reducing action that has been carried out in your group reduces your damage (if it occurs) by 4 taler, independent of whether you have taken such an action yourself. A damage-reducing action that you carry out costs you 5 taler for sure. In return, you reduce your damage and the damage of each other group member by 4 taler, if the damage occurs.

The personal damage, if it occurs, amounts to 20 taler if no one in your group carried out an action, 16 taler if one person carried out an action, 12 taler if two persons took the action, 8 taler if three persons took the action, and 4 taler if all group members took the action.

### Your round income (in taler) is calculated as follows

- If the damage does not occur and you did not take the damage-reducing action: 25
- If the damage does not occur and you did take the damage-reducing action: 25-5=20
- If the damage occurs and you did not take the damage-reducing action: 25-20+4∗ [sum of all damage-reducing actions in the group],
- If the damage occurs and you did take the damage-reducing action: 25-5-20+4∗ [sum of all damage-reducing actions in the group],

4 examples:

The damage probability always is 50%.

- You and one other group member take a damage-reducing action in your group, the damage does not occur. Your round income is 25-5=20 taler.
- Only you take a damage-reducing action in your group, the damage occurs. Your round income is 25-5-20+4∗1=4 taler.
- You and two other group members take a damage-reducing action, the damage occurs. Your round income is 25-5-20+4∗3=12 taler.
- Two other group members take a damage-reducing action, but you do not, the damage occurs. Your round income is 25-20+4∗2=13 taler.

At the end of a round, each participant receives information summarizing the player’s own decision, how many other group members took an action, if the damage occurred and what the round income is. Then, a new round starts in the same group constellation or in a new group constellation if a new phase begins.

The sum of all your round incomes will be paid out to you in private at the end of the experiment.

Before the experiment starts, we would like to ask you to answer some control questions on the computer to make sure you understand the rules.

## Footnotes

1 A famous example is given by the German policies following the nuclear reactor disaster at Fukushima, Japan: German former chancellor Angela Merkel said in March 2011 “The events in Japan mark a turning point for the world.” Another instance is increasing concern about climate change, expressed through the Fridays for Future movement in the aftermath of the record-setting hot summers of 2018 and 2019 (e.g., Smith, 2019).

2 Even if both channels are affected, actions may be viewed as affecting the probability of events of given magnitude or the magnitude of events of given probability depending on how changes in the distributions are communicated. McPhillips et al. (2018) discuss how the meaning of “extreme events” varies across disciplines, with some definitions focusing on the impact level and thus on actions as *changing* the probability, while others define extreme in the sense of probability/frequency (e.g., a 100-year flood), such that *changes* would be defined in terms of the magnitude.

3 Of course, expected payoffs conditional on the number of cooperators in the group are held constant across treatments. Notice that we test a fourth, additional treatment condition; its function becomes clear in the next section.

4 Earlier, Dickinson (1998) compares contributions in social dilemma games with probabilistic and certain gains and finds that risk decreases contributions. Related to this strand of the literature are studies on policy instruments for dealing with non-point source pollution (e.g., Segerson, 1988; Miceli and Segerson, 2007): in this case, fines can only be put on ambient pollution levels, triggering sanctions based on the group rather than individual behavior. Similarly, incentives for cooperative behavior in groups have been discussed in the context of industrial organization and team production (e.g., Holmstrom, 1982; Rasmussen, 1987; Varian, 1990).

5 Related to this, Muermann and Kunreuther (2008) provide theoretical evidence of individual under-investment for probability reduction of a stochastic loss (“self-protection”) when subjects participate from investments for probability reductions of others, too. Gong et al. (2009) explores the effect of within-group interaction versus between-group interactions on cooperation rates in the prisoner’s dilemma with stochastic payoffs. Determinants of altruistic decisions under risk are investigated, e.g., by Brock et al. (2013) and Freundt and Lange (2017).

6 Kandori (1992) demonstrates that there is no theoretical reason against the emergence of cooperative supergame equilibrium even under random matching. Dal Bò (2005) shows that behavior is more consistent with theoretical predictions in infinitely repeated two-player prisoner’s dilemma games compared to settings with finite repetitions. Tan and Wei (2014), and Lugovskyy et al. (2017) consider a multi-player social dilemma game and show a more pronounced decrease in cooperation in the finitely repeated setting in comparison to the setting with probabilistic ending.

7 In the experiment, we use neutral wording; the exact wording is “take/not take an action”.

8 In most settings, the chance for adverse events to occur is positive despite precautionary investments (e.g., security risk on airplane crashes).

9 An individual player, when cooperating, reduces the probability of the damage event by *x*. We implement this by a random device drawing a number *r* between 0 and 1, the damage occurs if the number is smaller than $p_0-xQ^t$. In our experimental setting, it is thus possible to define if a player’s decision is pivotal: $p_0-xQ_{-i}^t>r\ge p_0-x(Q_{-i}^t+1)$.

10 After each period, the supergame terminates with probability δ, and a new supergame starts in new randomly re-matched groups, whereas with probability 1-δ the supergame continues in the same group constellation. Playing across changing group compositions in supergames allows us to generate more observations per subject, thereby better accounting for potential learning behavior.

11 With this, our parameters satisfy the existence of a cooperative equilibrium which has been discussed by Dal Bò and Fréchette (2011) as a necessary condition for persistent cooperation in experiments.

12 While an experimental plan and hypotheses were formulated, the experiment was not pre-registered as pre-registration was not the standard procedure in 2014.

13 Throughout the paper and unless specified otherwise, statistical significance is assessed by two-sided Wilcoxon Mann-Whitney rank-sum tests relying on matching unit averages. Recall that the comparisons between *ProbRed* on the one side and *DamRed* and *DamRed1* on the other side are not straightforward, as there are several differences between the setups (i.e., the differences in quality of the feedback information and the different channels of cooperative protection). This is why we introduce the fourth treatment *ProbRed*$^+$. Nonetheless, we compare the treatment conditions statistically, as *ProbRed* and *DamRed* represent both reduction channels in isolation (reality is likely a combination of both). Comparing them will give us an indication of which channel is more promising for sustained cooperation.

14 According to F-tests, the time trend across supergames in a treatment (captured, by the marginal effect of *supergame* plus the interaction term *treatment* × *supergame*) is statistically different from zero for all treatments at p<0.05 except *ProbRed* (p=0.146). Similarly, the period effect within a treatment (*period in supergame* plus *treatment* × *period in supergame*) is statistically different from zero for all treatments at p<0.05.

15 Note that individual risk attitudes are measured after the main experiment. Yet, Appendix C shows that there is no indication that earnings or damage experiences affect the measured risk attitudes.

16 p<0.001 for both comparisons using two-sided Wilcoxon Mann–Whitney rank-sum tests, while the former two frequencies do not differ significantly (p=0.46)

17 This finding may be driven by the additional information that these players receive relative to *ProbRed*. For example, cooperators may learn in *ProbRed*$^{+}$ that they were not pivotal in preventing the damage and thus might be more willing to switch to defection than in *ProbRed*. We control for the distance between the actual cooperation level and the level needed to prevent the damage event in our following analysis.

18 Arellano–Bond is typically applied to continuous rather than discrete dependent variables. However, we are not aware of a fully consistent method that can both incorporate the lagged contribution variable and control for interdependencies at the individual and matching-unit levels. Our results, however, are robust to alternative specifications such as a random-effects probit model or OLS regressions with individually clustered errors.

19 This mechanism has prominently been formulated as the “Law of Effect” (dating back to Thorndike, 1898). It found its way into the behavioral economics literature (e.g., Selten and Stöcker, 1986) under different labels like reinforcement learning (e.g., Roth and Erev, 1995; Erev and Roth, 1998), experience-weighted attraction learning (e.g., Camerer and Ho, 1999; Ho et al., 2008), or impulse balance learning (e.g., Selten and Chmura, 2008; Chmura et al., 2012), to state the most prominent examples.

20 Notice that a similar logic can be applied when players anticipate regret (e.g., Loomes and Sugden, 1982; Zeelenberg, 1999; Filiz-Ozbay and Ozbay, 2007).

21 In contrast, the absence of a damage event may lead people to question whether the efforts were necessary. A version of this self-defeating prophecy was coined the “prevention paradox” during the COVID-19 pandemic. https://www.theguardian.com/world/2020/apr/26/virologist-christian-drosten-germany-coronavirus-expert-interview.
